# Associations between active video gaming and other energy-balance related behaviours in adolescents: a 24-hour recall diary study

**DOI:** 10.1186/s12966-015-0192-6

**Published:** 2015-03-05

**Authors:** Monique Simons, Mai JM Chinapaw, Johannes Brug, Jaap Seidell, Emely de Vet

**Affiliations:** Department of Health Sciences and the EMGO Institute for Health and Care Research, Faculty of Earth and Life Sciences, VU University Amsterdam, De Boelelaan 1085, 1081 HV Amsterdam, The Netherlands; Body@Work, Research Center Physical Activity, Work and Health, TNO- VU/VUmc, VU University Medical Center, Amsterdam, The Netherlands; TNO, Expertise Centre Life Style, Leiden, The Netherlands; Department of Public and Occupational Health and the EMGO Institute for Health and Care Research, VU University Medical Center, Amsterdam, The Netherlands; Department of Epidemiology & Biostatistics and EMGO Institute for Health and Care Research, VU University Medical Center, Amsterdam, The Netherlands; Chairgroup Strategic Communication, Sub-department Communication, Philosophy and Technology: Centre for Integrative Development, Wageningen University and Research Centre Wageningen, Wageningen, the Netherlands

**Keywords:** Active video games, Physical activity, Sedentary behaviour, Adolescents, Associations, Health behaviours, Snacks, Beverages, Energy intake

## Abstract

**Background:**

Active video games may contribute to reducing time spent in sedentary activities, increasing physical activity and preventing excessive weight gain in adolescents. Active video gaming can, however, only be beneficial for weight management when it replaces sedentary activities and not other physical activity, and when it is not associated with a higher energy intake. The current study therefore examines the association between active video gaming and other energy-balance-related behaviours (EBRBs).

**Findings:**

Adolescents (12–16 years) with access to an active video game and who reported to spend at least one hour per week in active video gaming were invited to participate in the study. They were asked to complete electronic 24-hour recall diaries on five randomly assigned weekdays and two randomly assigned weekend-days in a one-month period, reporting on time spent playing active and non-active video games and on other EBRBs. Findings indicated that adolescents who reported playing active video games on assessed days also reported spending more time playing non-active video games (Median = 23.6, IQR = 56.8 minutes per week) compared to adolescents who did not report playing active video games on assessed days (Median = 10.0, IQR = 51.3 minutes per week, *P* < 0.001 (Mann Whitney test)). No differences between these groups were found in other EBRBs. Among those who played active video games on assessed days, active video game time was positively yet weakly associated with TV/DVD time and snack consumption. Active video game time was not significantly associated with other activities and sugar-sweetened beverages intake.

**Conclusions:**

The results suggest that it is unlikely that time spent by adolescents in playing active video games replaces time spent in other physically active behaviours or sedentary activities. Spending more time playing active video games does seem to be associated with a small, but significant increase in intake of snacks. This suggests that interventions aimed at increasing time spent on active video gaming, may have unexpected side effects, thus warranting caution.

**Electronic supplementary material:**

The online version of this article (doi:10.1186/s12966-015-0192-6) contains supplementary material, which is available to authorized users.

## Findings

### Introduction

Replacing non-active video games (video games that are played sedentary) with active video games (video games that require physical activity to play) may contribute to reducing time spent in sedentary activities. Thus, physical activity may be increased and excessive weight gain in adolescents may be prevented [1,2]. Playing video games is a very popular activity among adolescents; an earlier study showed that the majority of Dutch adolescents play video games and almost half play active video games [3]. Interventions aimed at increasing physical activity through the promotion of active video gaming can therefore potentially have a wide reach. Furthermore, active video gaming seems to be particularly suitable for reaching adolescents with lower levels of education. Finally, it is at least equally suitable for reaching girls as it is for reaching boys [3,4].

It is, however, unclear how active video gaming is associated with other energy-balance related behaviours (EBRBs). This is important, as active video gaming may only be beneficial for energy balance when it replaces sedentary activities such as non-active video gaming or watching TV. When adolescents play active video games instead of other physically active activities such as outdoor play or sports, active video gaming will not contribute to increasing total energy expenditure [5,6]. Another important EBRB is the consumption of snacks and sugar-sweetened beverages. Studies on non-active video gaming show that non-active game time is associated with increased energy intake through snack or soft drink consumption [7-9]. It may be that active video gaming provides less opportunity for snacking because one is physically engaged in the game. On the other hand, two laboratory studies showed that consumption of snacks and sugar-sweetened beverages during active video gaming were equal to that during non-active video gaming. In addition, a study in adults compared energy intake during active video gaming and non-active video gaming (and watching TV) and did not find any significant differences [10], nor did a study in children that compared playing non-active games while sitting with playing non-active video games while using a treadmill [11]. However, these studies only focused on energy intake during video gaming and did not evaluate energy intake during the rest of the day. It may be that active video gaming induces the intake of snacks and sugar-sweetened beverages after the activity because of thirst and hunger associated with physical activity. ‘Real life’ studies are limited. Therefore, the current study explores ‘in real life’ to what extent active video game time is associated with time spent in other physically active behaviour, sedentary activities and the consumption of snacks and sugar-sweetened drinks.

### Method

A Dutch youth panel consisting of 2800 12-16-year-old adolescents with access to an active video game were invited to participate in the study and were screened for eligibility. This resulted in 596 adolescents who were willing to participate and met the criterion of spending at least one hour per week engaged in active video gaming. An active video game was defined as a video game that requires movement of the body, more than movement of the fingers and hands alone [12].

Adolescents were requested to complete electronic 24-hour recall diaries on five randomly assigned weekdays and two randomly assigned weekend days during a one-month period (April 2011), excluding holidays. On the assigned day, the adolescents were notified at 4 pm by a text message and an email which included web links to the diary. A reminder was sent the next morning at 8 am if the diary was not completed by that time. The diary could be completed until 9 am on the same day. Participants received a voucher worth 7.50 euro when all seven diaries were completed (in addition to the standard reward points system of the agency for each time they participate in a research).

**Figure 1:**
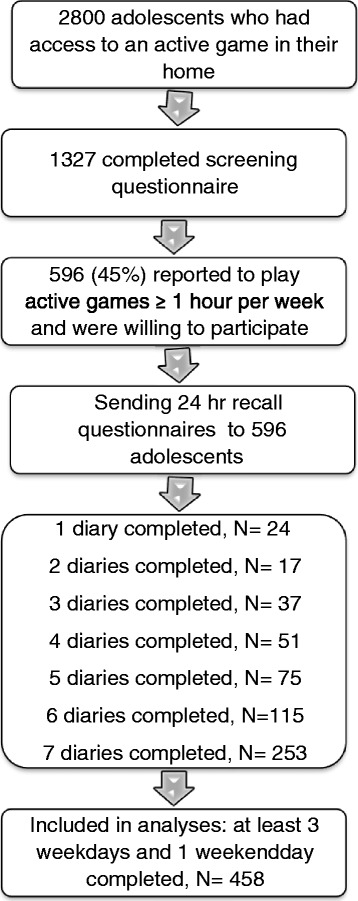
**Flow scheme.**

In total 458 participants completed their diary on at least three weekdays and one weekend day, which we considered a minimum for reliable estimates, and these were included in the analyses (see Figure 1 for flow scheme).

### Measurements

Demographic characteristics (age, gender, ethnicity and level of education) were assessed in the inclusion criteria screening questionnaire.

We developed an electronic 24-hour recall diary to assess engagement in and time spent on a range of EBRBs (Additional file 1). The diary used three time slots: 4:00 pm to bed time, rising time to 12:00 and 12.00 to 4:00 pm. For each time slot, adolescents were provided a list of 12 main categories of activities; 1) sleeping, 2) video gaming, 3) watching television (TV) or DVD, 4) non-video gaming computer (PC) use, 5) homework, 6) social activities, 7) other hobbies, 8) sports and active play, 9) walking and cycling, 10) other transportation, 11) household chores, and 12) work and internship. Adolescents were asked to select the activity they performed during the specific time slot and to report how many minutes they spent in that activity. In addition, the 24-hour recall diary assessed the quantity of snacks and sugar-sweetened beverages consumed during the same time slots. The mean number of minutes per day spent on each activity and mean intake per day were calculated. The questions in the diary were derived from validated questionnaires [13-17]. The questions to assess time spent playing video games were developed for the present study and are based on validated questionnaires for assessing time spent in sedentary activities [13,15,16]. The modified items were not further checked for validity.

### Data handling and statistical analyses

For each time slot we calculated the total time spent on activities by adding together all reported activities in that specific time frame. We used rise time and bedtime to calculate the total number of minutes awake in each time slot. We considered single activities exceeding the total amount of possible minutes within a specific time frame as unrealistic and treated them as missing values. Multi-tasking (i.e. more than one activity at the same time) was regarded as realistic, but when the different activities added up to more than double the available minutes in a specific time slot it was regarded as missing. Next, we categorized the activities into the following nine categories: 1) active video gaming, 2) non-active video gaming, 3) watching TV/DVD, 4) PC use other than video gaming, 5) non-screen sedentary activities, 6) sports and active play, 7) other physical activities (e.g. active transportation, household chores), 8) consumption of snacks and 9) consumption of sugar-sweetened drinks (Additional file 1).

We used non-parametric tests because the data was non-normally distributed. First, time use differences between adolescents who reported active video game play (active video gamers) at assessed days and adolescents who reported no active video game play at assessed days were tested with the Mann–Whitney test. Next, Spearman correlation coefficients were computed to evaluate associations between active video gaming time on the one hand and on the other hand non-active video gaming time, TV time, non-video gaming PC time, non-screen sedentary time, sports, other physical activities and consumption of snacks and sugar-sweetened drinks. The correlation coefficients were interpreted based on Cohen’s [18] guidelines (*r*⩾.50: strong association; .30⩽*r* < .50: moderate association; .10⩽*r* < .30: weak association). All statistical analyses were carried out using SPSS for windows, version 21.

## Results

### Results

The mean age of the participants was 13.7 years (SD = 1.4), 58% were male, 98% were of Dutch ethnicity, 9% attended primary school, 43% pre-vocational secondary education and 48% higher or –pre-university secondary education.

In comparison with adolescents who did not play active games on the assessed days, the active video gamers spent significantly more time on non-active video gaming (Mann–Whitney U = 19025.5; *P* < 0.001). Time spent in other activities and consumption of snacks and sugar-sweetened beverages did not differ between the two groups (Table 1). Among the active video gamers, active video gaming time was statistically, but only weakly, positively associated with TV/DVD time (r = 0.159, *P* = 0.047) and consumption of snacks (r = 0.168, *P* = 0.035). No associations were found between active video gaming and the other EBRBs (Table 2).

**Table 1:** **Median (IQR) for physical, sedentary and dietary behaviours among adolescents who did and did not play active video games at assessed days**

**Activity (min/day)**	**Adolescents who did play active video games (n = 157)**	**Adolescents who did not play active video games (n = 301)**	**Total group (n = 458)**	***P*** **value (Mann Whitney test)**
Active video gaming	13.3 (19.9)	0	0 (8.3)	NA
Non-active video gaming	23.6 (56.8)	10.0 (51.3)	15.0 (53.6)	<0.001
Watching TV/DVD	88.6 (81.4)	94.3 (92.7)	92.1 (86.2)	0.28
PC use other than video gaming	51.4 (71.2)	51.6 (69)	51.5 (70.5)	0.96
Non screen sedentary activities	111.4 (102.0)	110.0 (113.2)	110.4 (109.9)	0.99
Sports and active play	25.0 (51.3)	20.0 (54.6)	21.5 (54.6)	0.21
Other physical activity (>3 METS)	38.2 (49.3)	41.3 (52.0)	40.0 (51.1)	0.59
Snacking (servings/day)	3.1 (2.3)	3.0 (2.0)	3.0 (2.1)	0.73
Sugar-sweetened beverages (ml/day)	1107.1 (880.5)	1125.7 (971.7)	1116.6 (945.7)	0.53

IQR = interquartile range.

NA = not available.

**Table 2:** **Associations (Spearman correlations) and** ***P*** **-values of time spent in active video gaming with other energy balance related behaviours among active video gamers (N = 157)**

	**Spearman’s Rho**	***P*** **value**
**Non-active video gaming**	0.11	0.162
**Watching TV/DVD**	0.16^*a^	0.047
**PC use other than video gaming**	0.11	0.186
**Non-screen sedentary activities**	0.02	0.791
**Sports and active play (>3 METS)**	−0.03	0.680
**Other physical activity (>3 METS)**	0.07	0.359
**Consumption of snacks**	0.17*^a^	0.035
**Consumption of sugar-sweetened beverages**	0.07	0.417

*Correlation is significant at the 0.05 level (2-tailed).

^a^Small effect size, (Cohen [18]).

### Discussion and conclusion

Adolescents who reported playing active video games, also reported spending more time playing non-active video games than those who reported not to have played active video games. Other EBRBs did not differ between the two groups. These results suggest that an interest in active and non-active video gaming is associated, which may indicate that replacing non-active video game time for active video game time is possible. This is in line with an earlier study that showed that most adolescents who play active video games also play non-active video games [3]. However, it has also been suggested that adolescents who play video games prefer non-active video games over active video games [12] and that adolescents who play non-active video games differ from those who play active video games also in other respects [19]. These last two suggestions indicate that it may not be easy to motivate adolescents to replace non-active video gaming with active video gaming.

While active video gamers reported spending significantly more time on non-active video gaming than other adolescents, there was no significant association between the time spent on active video gaming and that spent on non-active video gaming time among the active video gamers. This may be a preliminary indication that the promotion of active video gaming would not result in a reduction of time spent on non-active video gaming. Support for this idea can be found in an earlier study that concluded that making active video games available in their home did not result in a decrease in self-reported non-active video game time [2]. Our results also suggest that engagement in active video game play is not associated with reduced time spent on (other) physically active behaviour. However, Baranowski et al. [20] suggest that children may compensate time spent on active video gaming by being less active at other times, because no change was found in total (objectively measured) physical activity in 9–12 year olds after making active video games available in their home. Finally, our results provide some reason for concern, because engagement in active video game play was significantly associated with more TV/DVD time, consistent with the findings of O’Loughlin et al. [4], and with a higher snack intake. Also, Lyons et al. found worrying findings regarding the energy balance; people who engaged in one hour of active video gaming consumed around 400 kcal more energy than they expended [10]. Although the energy surplus of active video gaming was 273 kcal less than the energy surplus for watching TV and non-active video gaming [10], caution is still warranted when using active video games in health promotion as such an intervention may have unexpected side effects.

A limitation of the current study is the self-report of behaviour, which is prone to socially desirable answers. The questions were based on validated questionnaires for assessing energy-balance related behaviours [13-17], but were modified to target video gaming behaviour and to make them suitable for online implementation. The modified items were not further checked for validity, which is another clear limitation of the present study. Furthermore, the study is cross-sectional and not causal and no causal inference can be made. We used 24-hour recall diaries, reducing recall bias compared to questionnaires recalling a previous or usual week. Also we assessed 7 random days within a month, providing more representative data than assessing only 1 or 2 days [21]. We did not study whether the interest in playing active video games was sustained during the month, which would be of interest for further research. Moreover, this is one of the first studies investigating the extent to which active video gaming is associated with other EBRBs in everyday life. Therefore, the current study provides very important insights into whether active video games can be an effective substitute for non-active video games in terms of preventing excessive weight gain. Nevertheless, more research is needed on the association between active video gaming and energy intake to get a clearer picture on what the beneficial and unanticipated effects of an active video gaming intervention can be. In addition, it would be of interest to compare active video gaming with other physical activities with regard to their respective associations with other EBRBs.

In summary, the results of our study suggest that it is unlikely that time spent playing active video games substitutes other physical or sedentary activities. However, spending more time playing active video games seems to be associated with a small, but significant increase in TV/DVD time and intake of snacks. Caution is therefore warranted in using active video games in health promotion, as such an intervention may have unexpected side effects.

## Additional file

Additional file 1:
**Overview of activities recalled in the 24 hours diary [** 13-17,22 **].**
