# Supplementary material for: Associations between active video gaming and other energy-balance related behaviours in adolescents: a 24-hour recall diary study
Source: Int J Behav Nutr Phys Act. 2015 Mar 5;12:32. doi: 10.1186/s12966-015-0192-6 (PMC4359402; doi:10.1186/s12966-015-0192-6)
Supplement: Additional file 1: — Overview of activities recalled in the 24 hours diary [ 13-17,22 ]. [file 12966_2015_192_MOESM1_ESM.docx]

**Additional file 1: Overview of activities recalled in the 24 hours diary**

| **Main activity as shown in diary** | **Question** | **Time frame** | **Activity category used for analyses (unit)** |
| --- | --- | --- | --- |
|  |  |  |  |
| 1. **Sleeping** |  |  |  |
| Bed time | What time did you go to bed? |  | **NA** |
| Rise time | What time did you got up? |  | **NA** |
| 1. **Video gaming** |  |  |  |
| **Active video gaming** | How long did you play active video games?  …hours and … minutes | 1. 4 pm - bed time 2. Rise time - noon 3. Noon till 4 pm | Active video gaming (Minutes/day) |
| **Non-active video gaming** | How long did you play non-active video games?  …hours and … minutes | 1. 4 pm - bed time 2. Rise time - noon 3. Noon till 4 pm | Non-active video gaming (Minutes/day) |
| 1. **Watching television or DVD** | How long did you watch TV/ DVD?  …hours and … minutes | 1. 4 pm - bed time 2. Rise time - noon 3. Noon till 4 pm | Watching TV/DVD (Minutes/day) |
| 1. **PC use other than video gaming** (surfing on the internet, watching YouTube movies, chatting etc) | How long did you use the computer?  …hours and … minutes | 1. 4 pm - bed time 2. Rise time - noon 3. Noon till 4 pm | Non-gaming PC use (Minutes/day) |
| 1. **Homework** | How much time did you spend on doing your homework?  …hours and … minutes | 1. 4 pm – bed time 2. Rise time – noon 3. Noon till 4 pm | Non-screen sedentary activities (minutes per day) |
| 1. **Social activities**  - phone calls - texting - chilling/hanging out with friends - going out - shopping | How long did you engage in the following activity?^2^  …hours and … minutes | 1. 4 pm – bed time 2. Rise time – noon 3. Noon till 4 pm | - <3 METS^4^ -> non-screen sedentary activities - >3 METS^4^ -> other physical activities |
| 1. **Other hobbies**  - Reading - Listening to music - Playing a musical instrument -> which instrument did you play? - Other sedentary activities (e.g. board games, drawing, crafting, puzzling) | How long did you engage in the following activity?^2^  …hours and … minutes | 1. 4 pm – bed time 2. Rise time – noon 3. Noon till 4 pm | - <3 METS^4^ -> non-screen sedentary activities - >3 METS^4^ -> other physical activities |
| 1. **Sports and active play** |  |  |  |
| - Soccer - Tennis - Swimming - Athletics - Hockey - Basketball - Martial arts (e.g. judo, karate, (kick)boxing) - Horse riding - Gymnastics - Ice skating - Dancing (e.g. Breakdance, salsa, ballroom dancing) - Fitness/aerobics - Skating - Running - Other, namely [OPEN answer] | How long did you engage in the following activity?^2^  …hours and … minutes | 1. 4 pm - bed time 2. Rise time - noon 3. Noon till 4 pm | Sport and active play (minutes/day) |
| 1. **Walking and cycling**  - to school/work/intern, - to (sport)club, union, friends, shops - just for leisure (e.g. cross around with your bike, walking your dog) | How much time did you spend on the following activity?^2^  …hours and … minutes | 1. 4 pm - bed time 2. Rise time - noon 3. Noon till 4 pm | Other physical activities |
| 1. **Other transportation** |  |  |  |
| Transportation by moped, car, public transportation (bus, train, metro, tram)   - to school/work/intern, - to the shops - other… | How much time did you spend on the following activity?^2^  …hours and … minutes | 1. 4 pm - bed time 2. Rise time - noon 3. Noon till 4 pm | Non-screen sedentary activities (minutes/day) |
| 1. **Household chores**   (e.g. cleaning up your room, doing the dishes, grocery shopping, cooking) | How much time did you spend on the following activity?^2^  …hours and … minutes | 1. 4 pm - bed time 2. Rise time - noon 3. Noon till 4 pm | Other physical activities (minutes/day) |
| 1. **Work and internship**  - Work - Internship | How much time did you spend on the following activity?^2^  …hours and … minutes  How would you the rate the intensity of the physical activity that is required for your work/internship?  Light, moderate, vigorous | 1. 4 pm - bed time 2. Rise time - noon 3. Noon till 4 pm | - <3 METS^4^ -> non-screen sedentary activities - >3 METS^4^ -> other physical activities |
| ***Energy intake^3^*** |  |  |  |
| **Consumption of snacks** | Number of servings (fried snacks, potato chips, candy bars) consumed between the main meals in the past 24 hours. | 1. 4 pm - bed time 2. Rise time - noon 3. Noon till 4 pm | Consumption of snacks (servings/day) |
| **Consumption of sugar-sweetened beverages** | (sport and energy drinks, non-carbonated and carbonated soda, excluding fruit juices)   - number of glasses consumed (200ml) - number of cans consumed (330ml) - number of bottles consumed (500ml). | 1. 4 pm - bed time 2. Rise time - noon 3. Noon till 4 pm | Consumption of sugar-sweetened beverages (ml/day) |

^1^Flemish Physical Activity Computer Questionnaires for adolescents [15], the Previous Day Physical Activity Recall (PDPAR) [13], and the Multimedia Activity Recall for Children and Adolescents (MARCA) [16] provided input for the questions on activities.

^2^Time expenditure was asked for each selected activity (see bullets in column ‘activity’) separately.

^3^Questions on energy intake were based on validated questionnaires of Van der Horst et al. [17] and van Assema et al. [14]

^4^According to compendium of Ainsworth et al., 2000 [22]
